# Effects of Acid Modulators on the Microwave-Assisted Synthesis of Cr/Sn Metal-Organic Frameworks

**DOI:** 10.3390/polym14183826

**Published:** 2022-09-13

**Authors:** Wei Mao, Renting Huang, Hao Xu, Hao Wang, Yi Huang, Shurong Huang, Jinghong Zhou

**Affiliations:** Guangxi Key Laboratory of Clean Pulp & Papermaking and Pollution Control, School of Light Industrial and Food Engineering, Guangxi University, Nanning 530004, China

**Keywords:** modulators, microwave synthesis, bimetallic MIL-101 (Cr, Sn)

## Abstract

Metal-organic frameworks (MOFs) have attracted remarkable attention for their distinguished structural designability. Precisely controlling the particle size and improving the structural stability of MOF nanoparticles influence their catalytic activity significantly. In this study, six acids (nitric, hydrochloric, formic, acetic, succinic, and citric acids) were used as modulators to prepare bimetallic MIL-101 (Cr, Sn) (MIL stands for Materials of Institut Lavoisier) via a microwave-assisted hydrothermal method. Changes in volumetric, structural, stability, and catalytic properties, size, and shape of MIL-101 (Cr, Sn) were examined using scanning electron microscopy, X-ray diffraction, thermogravimetric analysis, and N_2_ adsorption–desorption measurements. All modulators altered the MOF properties. Compared with other samples, acetic acid as a modulator mildly altered the MOF morphology by narrowing their particle size distribution, enhancing the specific surface area, and significantly improving their water and thermal stabilities. The addition of acetic acid was suitable for the catalytic conversion of glucose to 5-hydroxymethylfurfural (5-HMF), achieving a 43.1% 5-HMF yield with 91.4% glucose conversion in a mixed solution of γ-valerolactone and saturated salt water at 150 °C after 30 min.

## 1. Introduction

Metal-organic frameworks (MOFs) have attracted increasing attention worldwide for their diversity, tuneability, and highly porous structure, which provide great potential for application [1]. MOFs consist of metal clusters and charged organic ligands [1,2] that can be synthesized following the principle of network chemistry to form strong bonds [3,4]. Through the flexible selection of metal clusters and organic components, the crystal structure and physiochemical properties of MOFs can be deliberately designed, rendering them an interesting platform for specific purposes [5,6,7], such as catalysis [8,9], adsorption [10], separation [11], sensing [12], and storage [13].

Hao et al. [14] synthesized bimetallic MIL-101 (Cr, Sn) with more crystal defects by in situ doping of Sn^4+^ into MIL-101 (Cr), demonstrating low loading and high dispersion of Sn doping. In addition to metal ions and organic ligands, diverse MOF materials can also be used to obtain desired properties by defect engineering. The most common method for controlling this defect is to add modulators. Zhou et al. [15] used acetic acid (AA) as a modulator in the synthesis of MOFs and found that the porosity of UiO-66 (UiO stands for University of Oslo) tended to increase with increasing amounts of acetic acid. The acidity of the modulator also strongly influences the nature of defects in the UiO-66 framework [16]. Bagherzadeh et al. [17] investigated the effects of AA and formic acid (FA) as modulators on the size and morphology of MIL-88A particles during the formation process. They found that adding modulators can either promote the nucleation process or interfere with the growth of particles. Cai and Jiang [18] found that introducing AA, octanoic acid, and dodecanoic acid during synthesis pre-occupies the ligand site, causing structural defects through incomplete ligand exchange. Similar conclusions were drawn by Wang et al. [19], who synthesized defective UiO-66 samples using trifluoroacetic acid and FA as modulators, and found that the catalytic performance of UiO-66 was improved.

The modulator is usually a monocarboxylic acid [20,21], which competes with organic ligands in the synthesis of MOFs and coordinates with metal sites to produce structural defects, leading to chemical bond breakage and lattice distortion, ultimately enhancing catalytic performance [22,23,24]. Currently, the most common MIL-101 (Cr) synthesis method uses hydrofluoric acid (HF) as a modulator to improve crystallinity and porosity. Zhao et al. [25] synthesized MIL-101 (Cr) using HF as a modulator by hydrothermal heating at 220 °C for 8 h and washing with *N,N*-dimethylformamide and ethanol for several hours, achieving a yield of about 50% and a specific surface area of 2400–3500 m^2^ g^−1^.

In recent years, microwave irradiation has been used to synthesize inorganic nanomaterials, such as zeolite and MOFs [26,27,28]. During synthesis with the classical solvothermal method, thermal energy is transferred from the heat source to the solution via the reaction vessel [29,30], which is time-consuming, generally requiring several hours to several days [31,32]. However, irradiation directly interacts with the reactants during the MW synthesis, resulting in more efficient and faster heating. Jhung et al. [33] reported the synthesis of MIL-100 in the presence of HF at 220 °C in a microwave oven. The results showed that the crystal yield was 44% after 4 h of reaction, while the crystal yield after 4 days of conventional solvothermal synthesis was 45%. Choi et al. [34] also used microwaves to synthesize MOF-5, achieving crystal formation after 30 min of reaction, while the conventional solvothermal required 24 h.

One interesting area in the microwave-assisted synthesis of MOFs is defect engineering. Babarao et al. [35] synthesized UiO-66 crystals with different defect concentrations using hydrochloric acid and FA as modulators by microwave solvothermal synthesis. They studied the correlation between the composition of defect concentration and its carbon dioxide adsorption characteristics. Although many methods have been developed for synthesizing MOFs in hydrothermal kettles using modulators, to the best of our knowledge, there have been no studies on the simultaneous synthesis of MIL-101 (Cr, Sn) using modulator-assisted microwave hydrothermal methods. In this study, we systematically investigated the roles of strong acids containing N elements of the same period as F elements and Cl elements of the same main group (i.e., nitric acid (NA) and hydrochloric acid (HA)) as inorganic acid modulators, as well as monocarboxylic acids FA and AA with different carbon chain lengths, and succinic acid (SA) and citric acid (CA) with different numbers of carboxyl groups as organic acid modulators, in the crystal morphology, particle size, and other properties of MIL-101 (Cr, Sn).

## 2. Materials and Methods

### 2.1. Materials

Chromium (III) nitrate nonahydrate (Cr(NO_3_)_3_·9H_2_O, 99%), anhydrous tin (IV) chloride (SnCl_4_), terephthalic acid (H_2_BDC, 99%), 5-hydroxymethylfurfural (5-HMF, 99%), d-glucose (99%), γ-valerolactone (GVL, 98%), and methanol were purchased from Shanghai Aladdin Biochemical Technology Co., Ltd. (Shanghai, China). Sulfuric acid (98%), HA (37%), FA (≥88%), CA (≥99.5%), SA (≥99%), and AA (≥99.5%) were purchased from Tianjin Zhiyuan Chemical Reagent Co., Ltd. (Tianjin, China). NA (65–68%) and sodium chloride (NaCl, ≥99.5%) were purchased from Tianjin Damao Chemical Reagent Factory (Tianjin, China). Methanol was of chromatographic grade, and all other reagents were of analytical reagent (AR) grade and were used without further purification.

### 2.2. General Methods

The powder crystal structure of the samples was measured through powder X-ray diffraction (PXRD) using MINIFLEX 600 (Rigaku, Tokyo, Japan) with a flat sample holder. The specific surface area, pore volume, and pore size distribution of the prepared samples were determined using ASAP-2460 (Micromeritics, Norcross, GA, USA). A Sigma 300 (Zeiss, Oberkochen, Germany) scanning electron microscope (SEM) and JEM F200 (JEOL Ltd., Tokyo, Japan) transmission electron microscope (TEM) were used for the morphological characterization of the samples. The average particle size was calculated by randomly selecting 100 particles using the ImageJ 1.52a software (Wayne Rasband, National Institutes of Health, Bethesda, MD, USA) and Gaussian fitting. The catalytic products were analyzed using 1260 Infinity II series high-performance liquid chromatography (HPLC; Agilent Technologies, Santa Clara, CA, USA) on an Aminex@HPX-87H column (7.8 mm × 300 mm; Bio-Rad Laboratories, Hercules, CA, USA) and a ZORBAX Eclipse XDC-C18 column (4.6 mm × 250 mm; Agilent Technologies) equipped with a UV detector and a differential refractive index detector (Agilent Technologies, Santa Clara, CA, USA).

### 2.3. Material Synthesis

Diagram for the synthesis studies along with ligand structure and modulators of MIL-101 (Cr, Sn) are shown in Figure S1 (see Supplementary Materials). We added 3.5 mmol of Cr(NO_3_)_3_·9H_2_O, 0.5 mmol of SnCl_4_, 32 mmol of AA, and 4 mmol of H_2_BDC to deionized water (24 mL, 1.32 mol). At this stage, all solutions appear to be dark purple (Figure S2, see Supplementary Materials). The mixture was stirred for 30 min at 25 °C and then uniformly transferred to the polytetrafluoroethylene tank (100 mL) of the ATPIO-6T microwave hydrothermal parallel synthesizer (Nanjing Xian’ou Instrument Manufacturing Co., Ltd., Nanjing, China). The microwave power was set to 800 W, and the temperature was increased to 220 °C for 10 min, followed by a constant-temperature reaction for 60 min. After the reaction was completed, it was allowed to cool naturally to room temperature; then, the reaction solution was transferred to a centrifuge tube and centrifuged at 8000 rpm for 15 min. After centrifugation, the pellet containing green crystals was put into the microwave reaction tank, DMF (30 mL) was added, the power was set to 800 W, the temperature was increased to 100 °C for 5 min, and the reaction was performed at a constant temperature for 30 min. After cooling to room temperature, the supernatant was discarded, and the sample was washed by adding anhydrous ethanol (30 mL) under the above conditions; this operation was repeated one time. After the final centrifugation, the sample was dried overnight (12 h) in an oven (150 °C); the crystal powder showed different bright and dark green colors after grinding. Similarly, MIL-101 (Cr, Sn) was synthesized using six different acid modulators (4 mmol), such as nitric (NA), hydrochloric (HA), formic (FA), succinic (SA), citric acids(CA), and blank sample (without modulators).

### 2.4. Stability Analysis

Chromium ion dissolution analysis of the catalysts was performed using a contrAA 800 D (Analytik Jena, Jena, Germany) graphite furnace atomic absorption spectrometer (AAS) at 25 °C to characterize the water stability of the samples. The catalyst (50 mg) was placed in a screw-capped glass tube, mixed with 40 mL of aqueous solution at pH 2 and 7, and allowed to stand at room temperature. The supernatant was collected on days 1, 3, and 5 and filtered through a 0.22 μm filter membrane to determine the chromium ion content by AAS. The thermal stability of the catalysts under an air atmosphere was measured using the STA 449F5 (NETZSCH, Selb, Germany) thermogravimetric analyzer (TGA). The temperature range was 30–850 °C with a ramp rate of 10 °C/min.

### 2.5. Contact Angle Measurements

Samples (100 mg) were compacted into a circular sheet at 4 psi for 2 min using a compaction densitometer (Carve, Wabash, IN, USA). A drop of distilled water (2 μL) was slowly poured on the sample using a micro syringe. The contact angles of the samples were measured using a DSA30S (KRÜSS Scientific, Hamburg, Germany) contact angle meter.

### 2.6. Catalytic Experiment

For catalytic experiments, 18 mL GVL and 2 mL of saturated NaCl solution were used as the extraction and reaction phases, respectively. We used 150 mg of MIL-101 (Cr, Sn) and 60 μL of concentrated sulfuric acid as the catalyst, and 150 mg of glucose as the substrate. The reaction mixture was placed in the same polytetrafluoroethylene tank (100 mL) and sealed. The microwave power was set to 800 W, and the temperature was increased to 150 °C for 5 min, followed by a constant-temperature reaction for 30 min. The reaction tank was cooled to room temperature at the end of the reaction. The solid catalyst was removed by centrifugation, and the filtered solution was determined using HPLC for 5-HMF, fructose, and the by-products FA and levulinic acid (LA).

### 2.7. Product Analysis

Fructose, FA, and LA were detected using a differential refractive index detector with the AminexHPX-87H column operating at 60 °C and a detector operating at 40 °C. The mobile phase was a 5 mmol/L sulfuric acid solution at a 0.6 mL/min flow rate. Glucose conversion was calculated based on carbon content. The 5-HMF was detected using a UV variable-wavelength detector on a ZORBAX Eclipse XDC-C18 column with a detection wavelength of 285 nm and an operating temperature of 30 °C for the column and 40 °C for the detector. The mobile phases were 1% glacial AA solution and pure methanol solution, mixed at 90:10 (*v*/*v*) using a quadruplex pump at a 1.0 mL/min flow rate. The sample injection volume was 20 μL. The mobile phase and samples were filtered through 0.22 μm organic or aqueous filter membranes. Glucose conversion (%), 5-HMF yield (mol%), fructose yield (wt%), FA yield (wt%), and LA yield (wt%) were calculated as follows:

Glucose conversion (%):

(1) $${\mathrm{Glucose}}_{\mathrm{conversion}}=\left(1-\frac{\mathrm{mass}\;\mathrm{of}\;\mathrm{remaining}\;\mathrm{glucose}}{\mathrm{mass}\;\mathrm{of}\;\mathrm{starting}\;\mathrm{glucose}}\right)\times 100\%$$

5-HMF yield (mol%) and selectivity (%):

(2) $${\mathrm{5-HMF}}_{\mathrm{yield}}=\frac{\mathrm{moles}\;\mathrm{of}\;\mathrm{5-HMF}\;\mathrm{in}\;\mathrm{solution}}{\mathrm{moles}\;\mathrm{of}\;\mathrm{glucose}\;\mathrm{in}\;\mathrm{the}\;\mathrm{reaction}}\times 100\%$$

(3) $${\mathrm{5-HMF}}_{\mathrm{selectivity}}=\frac{{\mathrm{5-HMF}}_{\mathrm{yield}}}{{\mathrm{Glucose}}_{\mathrm{conversion}}}\times 100\%$$

Fructose yield (wt%), FA yield (wt%), and LA yield (wt%):

(4) $${\mathrm{Fructose}}_{\mathrm{yield}}=\frac{\mathrm{mass}\;\mathrm{of}\;\mathrm{remaining}\;\mathrm{fructose}}{\mathrm{mass}\;\mathrm{of}\;\mathrm{starting}\;\mathrm{glucose}}\times 100\%$$

(5) $${\mathrm{FA}}_{\mathrm{yield}}=\frac{\mathrm{mass}\;\mathrm{of}\;\mathrm{remaining}\;\mathrm{FA}}{\mathrm{mass}\;\mathrm{of}\;\mathrm{starting}\;\mathrm{glucose}}\times 100\%$$

(6) $${\mathrm{LA}}_{\mathrm{yield}}=\frac{\mathrm{mass}\;\mathrm{of}\;\mathrm{remaining}\;\mathrm{LA}}{\mathrm{mass}\;\mathrm{of}\;\mathrm{starting}\;\mathrm{glucose}}\times 100\%$$

## 3. Results and Discussion

**Figure 1:**
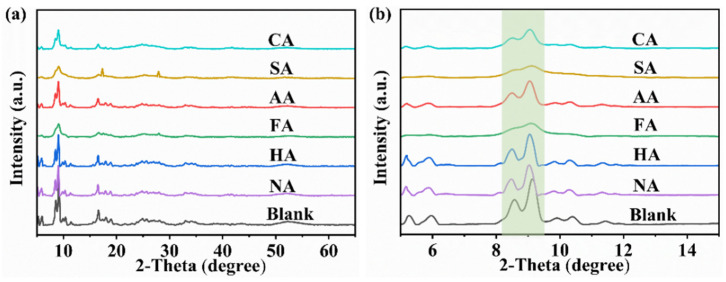
PXRD patterns of the blank sample and MIL-101 (Cr, Sn) synthesized with different modulators. (**a**) 5–65° and (**b**) 5–15°.

**Figure 2:**
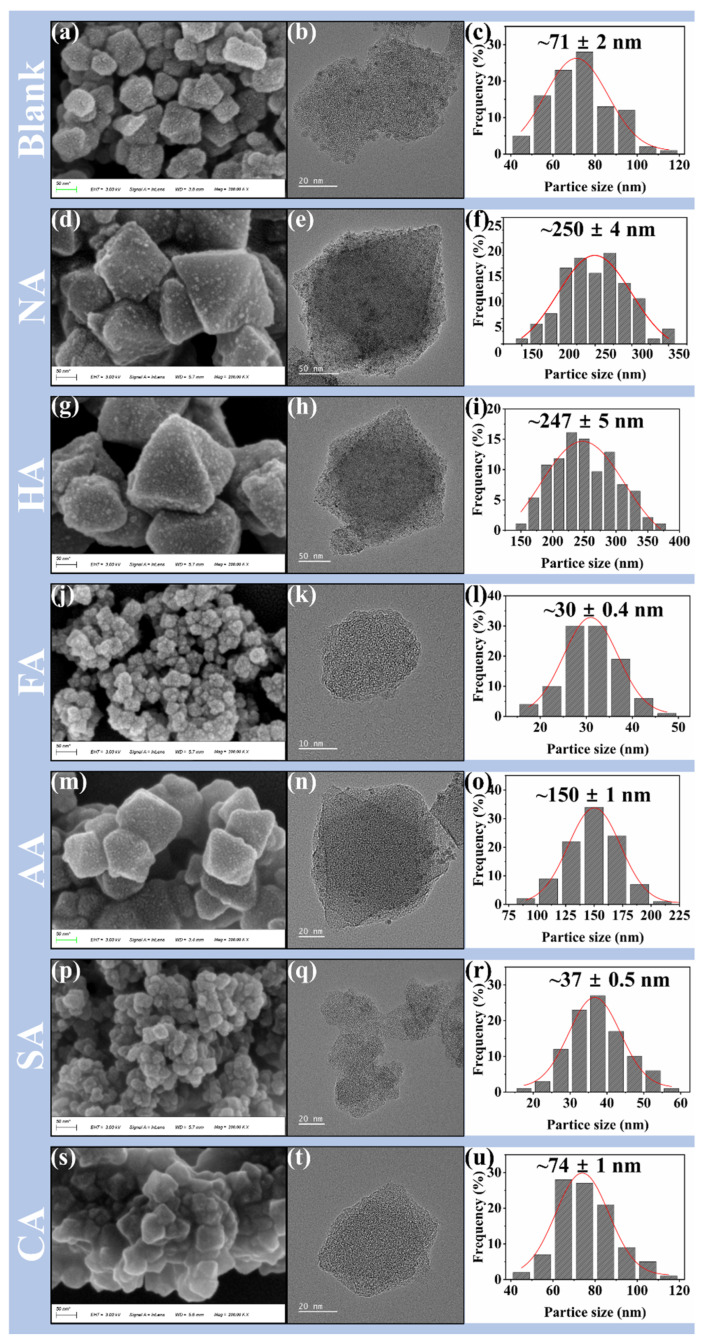
SEM (scale bar is 50 nm) images, TEM images, and particle size distribution images of (**a**–**c**) blank sample, (**d**–**f**) nitric acid, (**g**–**i**) hydrochloric acid, (**j**–**l**) formic acid, (**m**–**o**) acetic acid, (**p**–**r**) succinic acid, and (**s**–**u**) citric acid.

The crystal structures of the seven samples were determined using the complete set of PXRD patterns. As shown in Figure 1a,b, the characteristic diffraction peaks of blank sample and MIL-101 (Cr, Sn) with acid modulators appeared at 5.2°, 5.9°, 8.5°, and 9.0° [14]. Compared to blank samples, the main characteristic peak intensities of the samples synthesized by adding an acid modulator decreased. MIL-101 (Cr, Sn) synthesized with FA and SA as modulators showed a broad peak at the same position, no longer exhibiting good characteristic peaks. Moreover, the addition of these two acid modulators led to a smaller particle size (Figure 2j,p) and relatively narrow particle size distribution of MIL-101 (Cr, Sn) (Figure 2l,r), which was further confirmed by SEM analysis. Due to the fast heating, the microwave hydrothermal method rapidly consumes precursors, achieving rapid nucleation of crystalline MOFs [1]. However, the addition of acid modulators interferes with crystallization, resulting in the generation of small-particle aggregates and the destruction of the crystal structure.

The control of the size and morphology of MOFs is of great significance for their practical applications. Compared with the commonly used hydrothermal method, MIL-101 synthesized by the microwave-assisted hydrothermal method showed a smaller particle size, generally up to the nanometer scale [36]. Figure 2 shows the SEM images of the blank sample and MIL-101 (Cr, Sn) synthesized using different acid modulators. The addition of different acid modifiers changed the morphology and particle size of MIL-101 crystals [37]. The particle size distribution of the blank sample (Figure 2a–c) was between 40 and 120 nm, with an average particle size of 71 nm. Kulkarni et al. [38] used the hydrothermal method to obtain MIL-101 with a particle size distribution of 100–250 nm after 8 h of reaction at 220 °C without an acid modulator. Thus, rapid heating caused by the microwave hydrothermal method can produce nanoparticles with small particle sizes and relatively narrow size distribution [39]. Adding NA (Figure 2d–f) and HA (Figure 2g–i) resulted in better crystallinity; the obtained crystals generally showed a uniform and complete octahedral shape. The particle size was broadly distributed between 150–400 nm, with an average particle size of 250 nm. In contrast, adding FA (Figure 2j–l) resulted in the agglomeration of small particles with irregular crystal shapes. The particle size distribution was narrow between 15–50 nm, with an average particle size of 30 nm. The synthesis of MIL-101 (Cr, Sn) with SA (Figure 2p–r) as a modulator resulted in crystals with an irregular partial agglomeration of small particles and interconnected lamellar structures, with a particle size distribution between 10–60 nm and an average particle size of 37 nm. Adding AA (Figure 2m–o) and CA (Figure 2s–u) yielded crystals with mixed octahedral and irregular shapes. The average particle size of the crystals produced after adding AA was 150 nm, whereas after adding CA it was 74 nm. These results indicated that different modulators greatly influence the morphology and particle size of MOFs crystals. MIL-101 (Cr, Sn) modulated by FA or SA can result in uniform crystals with a smaller particle size.

**Figure 3:**
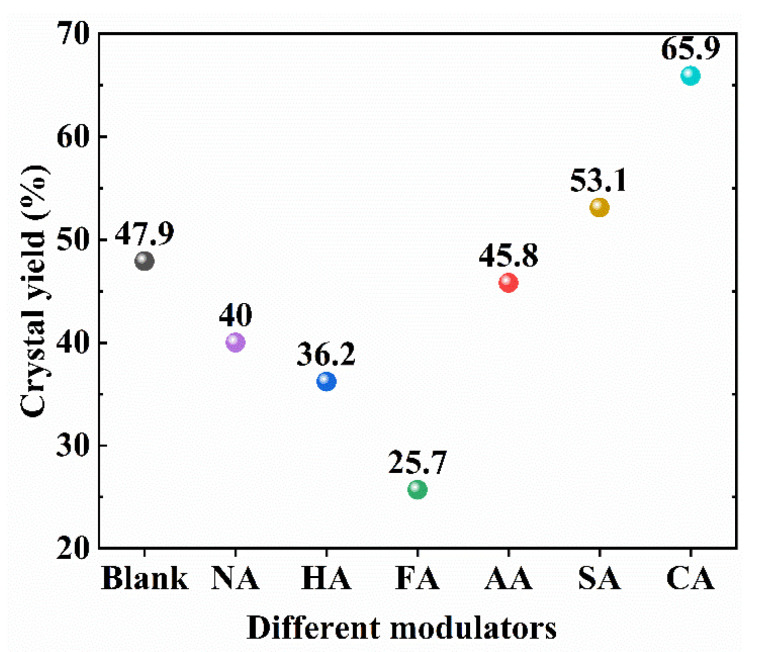
Crystal yield of the blank sample and MIL-101 (Cr, Sn) modulated by different acid modulators.

In blank samples, the addition of acid modulators differentially affected the crystal yield (Figure 3). In particular, the yield of MIL-101 (Cr, Sn) modulated by FA was the lowest (25.7%), whereas that of MIL-101 (Cr, Sn) modulated by CA was the highest (65.9%). The yield of MIL-101 (Cr, Sn) modulated by AA was 45.8%, consistent with a previous study. MIL-101 (Cr, Sn) modulated by HA was concomitant with chloride ions introduced into nodes as charge compensators [39], reducing the crystal yield to 36.2%.

**Figure 4:**
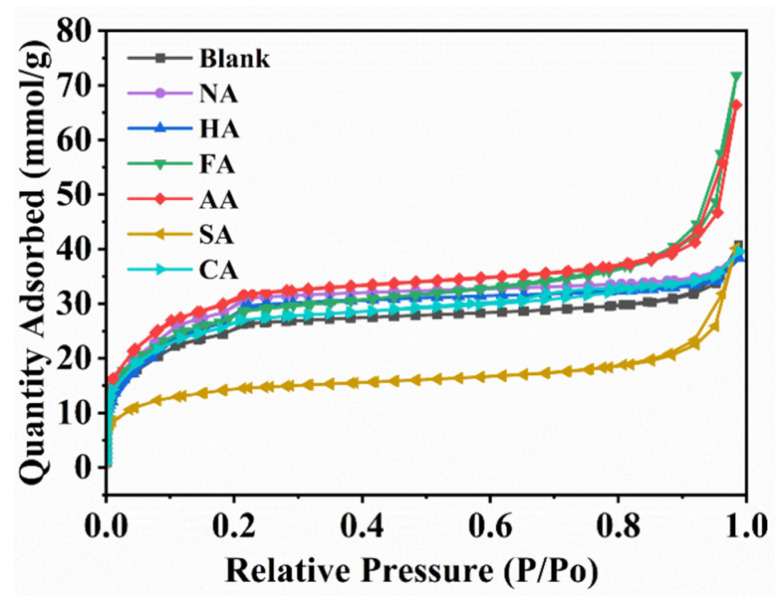
Nitrogen adsorption–desorption isotherms of the blank sample and MIL-101 (Cr, Sn) synthesized with different modulators.

The specific surface area (SSA) and pores of MOF materials play a crucial role in catalysis, effectively promoting the contact of reactants with the active sites on the MOF surface and improving their catalytic performance [40]. To investigate the effect of modulators on porosity, the nitrogen adsorption–desorption isotherms of all samples were measured (Figure 4), and SSAs were calculated according to the Brunauer-Emmett-Teller (BET) model (Table 1). The adsorption isotherm was a typical type I adsorption isotherm belonging to microporous materials; its hysteresis loop can be attributed to the whole adsorption between materials. SSA, pore size, and pore volume correlated with the type of modulator used in the synthesis. The sample with AA had the largest SSA (2504 m^2^ g^−1^), whereas that with SA had the smallest SSA (1160 m^2^ g^−1^). The SSA of other modulators was within 2100–2500 m^2^ g^−1^, pore size was 0.76–0.92 nm, and pore volume was 0.44–0.51 cm^3^ g^−1^. The addition of acid modulators, except SA, increased the SSA of MIL-101 (Cr, Sn) to varying degrees.

**Figure 5:**
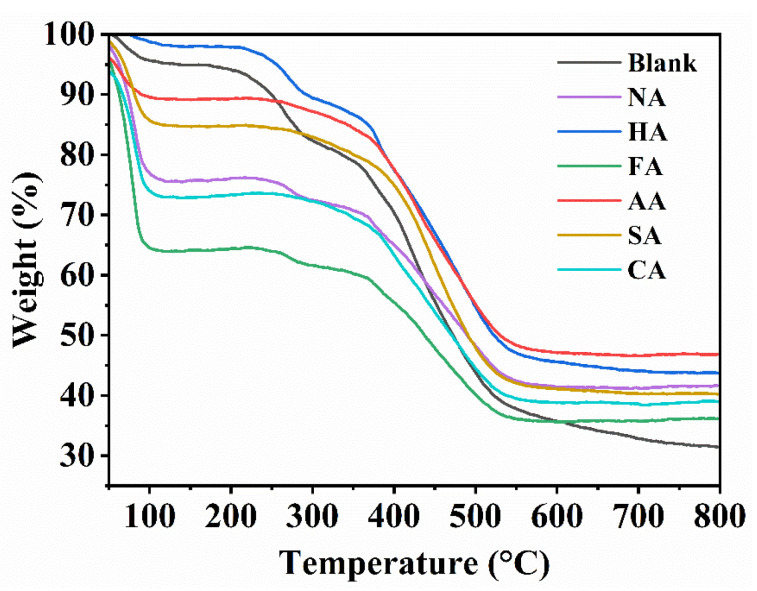
TGA curves of the blank sample and MIL-101 (Cr, Sn) synthesized with different modulators.

Good hydrothermal stability is essential for MOFs to ensure long-lasting and effective catalytic effects. MIL-101 (Cr) is one of the few MOFs with good hydrothermal stability up to 300 °C in air and 7 d in boiling water at 100 °C [41]. The TGA curves revealed the structural thermal stability of the blank sample and MIL-101 (Cr, Sn) with the addition of different modulators (Figure 5). The removal process of physically adsorbed free water occurred before 100 °C. The difference in the mass loss at this stage was due to the addition of different conditioners, resulting in different adsorption capacities of MIL-101 (Cr, Sn) to water vapor in the air. At this stage, the water loss mass of the blank sample was 4.38%. MIL-101 (Cr, Sn) synthesized with FA revealed a maximum water loss mass of 36.46%, and MIL-101 (Cr, Sn) synthesized with HA had the smallest water loss mass of 1.72%. The water loss mass of the other modulators ranked in the order AA < SA < NA < CA, ranging from 10.46% to 25.91%. Furthermore, 100–250 °C was stable, and 250–400 °C caused the removal of hydroxyl groups from the structure [42]. H_2_BDC decomposed between 400–500 °C, and the framework structure began to collapse, leading to a rapid decrease in mass. Between 30–800 °C, the maximum mass loss of the blank sample was 68.31%, that of MIL-101 (Cr, Sn) synthesized with AA was 53.98%, and that of MIL-101 (Cr, Sn) synthesized with other acids were 57.08–63.76%.

This result indicates that the addition of regulators increased the thermal stability of MIL-101 (Cr, Sn). It can be seen that MIL-101 (Cr, Sn) synthesized with FA had a relatively large pore size and pore volume, yielding more hydrophilic sites with a maximum water loss mass. MIL-101 (Cr, Sn) synthesized with HA showed the smallest pore size, pore volume, and water loss mass. Although MIL-101 (Cr, Sn) synthesized with SA as modulator had the largest pore structure, it also had the smallest SSA, with a water loss mass close to the average of all samples. While the SSA, pore size, and pore capacity of MIL-101 (Cr, Sn) synthesized with AA and NA as modulators were similar, the water loss mass MIL-101 (Cr, Sn) synthesized with AA was significantly smaller than that of MIL-101 synthesized with NA. We believe that this counterintuitive result may be attributable to the average particle size of AA (150 nm) being smaller than that of NA (250 nm), allowing water molecules to easily enter NA and be adsorbed.

**Figure 6:**
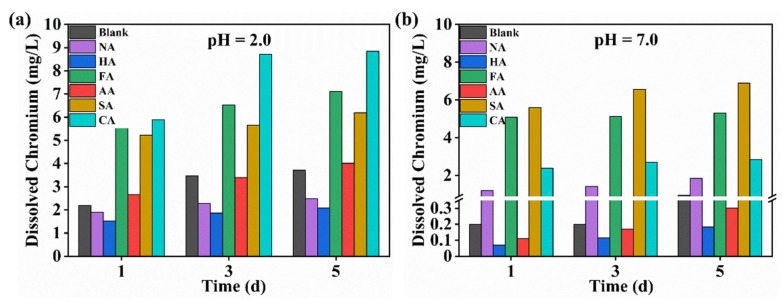
Variation in dissolved chromium ions with time for the blank sample and MIL-101 (Cr, Sn) synthesized with different modulators at different pH solutions; (**a**) pH 2, (**b**) pH 7.

Generally, the catalytic conversion of 5-HMF is performed in various solvent environments, especially acidic. However, in aqueous environments, the organic ligands of MOFs are readily substituted by water molecules [43]. MOFs are formed through coordination bonds between organic ligands and metal clusters, which leads to their instability in acidic and neutral solutions. The chemical bond between the metal ion and organic ligand is broken, eventually leading to the collapse of the skeleton [44]. The water stability of MOFs can be demonstrated through metal ion leaching in the solution. We illustrate the water stability of MIL-101 (Cr, Sn) by the amount of dissolution rate of Cr ions (Figure 6) [45]. The change in Cr ion leaching during immersion in solutions with different pH was examined to investigate the effect of modulators on the water stability of MIL-101 (Cr, Sn). However, as the dissolution rate of Cr ions was undetectable in a short time after MOF was added to different pH solutions due to the limit of detection of the instrument, we could only measure specific values starting from the first day after adding the solution.

In a water stability experiment of MIL-101 (Cr), Qiu et al. [45] mixed 1 g of sample with 50 mL of distilled water and left it at room temperature. After treatment with pH 2 solutions, the amount of Cr ion leaching in MIL-101 (Cr) was about 11, 27, and 36 mg/L on days 1, 3, and 5, respectively, and at pH 7, the amount of Cr ion leaching on days 1, 3, and 5 was about 5, 13, and 22 mg/L, respectively. In our experiments, the mass of tested samples was reduced by a factor of 20 compared to the results of Qiu et al. However, relative to the blank sample, after treatment with pH 2 solutions, the amount of Cr ion leaching in MIL-101 (Cr, Sn) was 2.19 mg/L, 3.46 mg/L, and 3.71 mg/L on days 1, 3, and 5, respectively, and at pH 7, the amount of Cr ion leaching on days 1, 3, and 5 was 0.2 mg/L, 0.2 mg/L, and 0.96 mg/L, respectively. When FA, SA, and CA were used as modulators, the total Cr ion leaching concentration increased more than two times compared to that without modulators. Compared to MIL-101 (Cr, Sn) modulated by FA, SA, and CA, MIL-101 (Cr, Sn) modulated by NA, HA, and AA exhibited good stability in aqueous solutions of different pH, soaking time, and solution pH, presumably because the dissolution rate of Cr ions is relatively low compared to that of other modulators.

**Figure 7:**
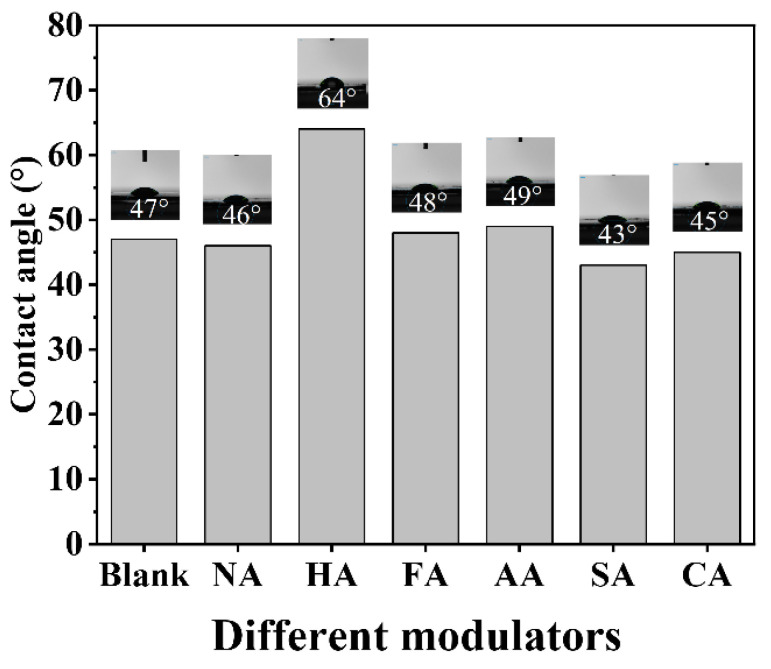
Contact angles of the blank sample and MIL-101 (Cr, Sn) synthesized with different modulators.

In general, hydrophobic MOFs exhibit higher catalytic activity and selectivity in catalysis. The hydrophobic UiO-66-SO_3_H synthesized by Li et al. reached a contact angle of 129° with distilled water. The yield of 5-HMF reached 98% in a dimethyl sulfoxide solution environment at 120 °C [46]. Based on these considerations, we conducted contact angle measurements; the results are shown in Figure 7. MIL-101 (Cr, Sn) modulated by HA had a maximum contact angle of 64° and the lowest Cr ion leaching rate in solutions with different pH compared to the blank and other samples. MIL-101 (Cr, Sn) modulated by AA had a contact angle of 49°. The contact angles of MIL-101 (Cr, Sn) modulated by SA and CA were 43° and 45°, respectively, and they also exhibited relatively high Cr ion solubilization rates. The contact angle of MIL-101 (Cr, Sn) modulated by other acids did not change significantly compared with the blank sample, except for MIL-101 (Cr, Sn) modulated by HA.

The MIL-101 (Cr, Sn) that we synthesized was defective, yielding unsaturated coordination of the metal center, which strongly interacts with water, making the material less hydrophobic; all samples were hydrophilic. MIL-101 (Cr, Sn) synthesized with HA achieved the maximum contact angle, possibly due to Cl ion coordination with the metal, producing saturation sites that enhance the hydrophobic properties and increase the contact angle. Organic ligands containing hydrophobic groups significantly enhance the contact angle of MOFs. While –COOH groups are hydrophilic, excessive –COOH groups reduce the hydrophobic properties of the material, decreasing the contact angle. In addition, since H_2_BDC in equal molar amounts with metals as organic ligands is already present in excess, AA, CA, and SA can hardly participate in coordination as modulators, causing slight differences in their hydrophobic properties. Considering the experimental error factor, the contact angle results are consistent with that of the dissolution rate of Cr ions in solutions with different pH; as the contact angle of the sample increases, its hydrophobic property proportionally increases, achieving better stability in different pH aqueous solutions, with an accordingly reduced Cr ion dissolution rate.

**Figure 8:**
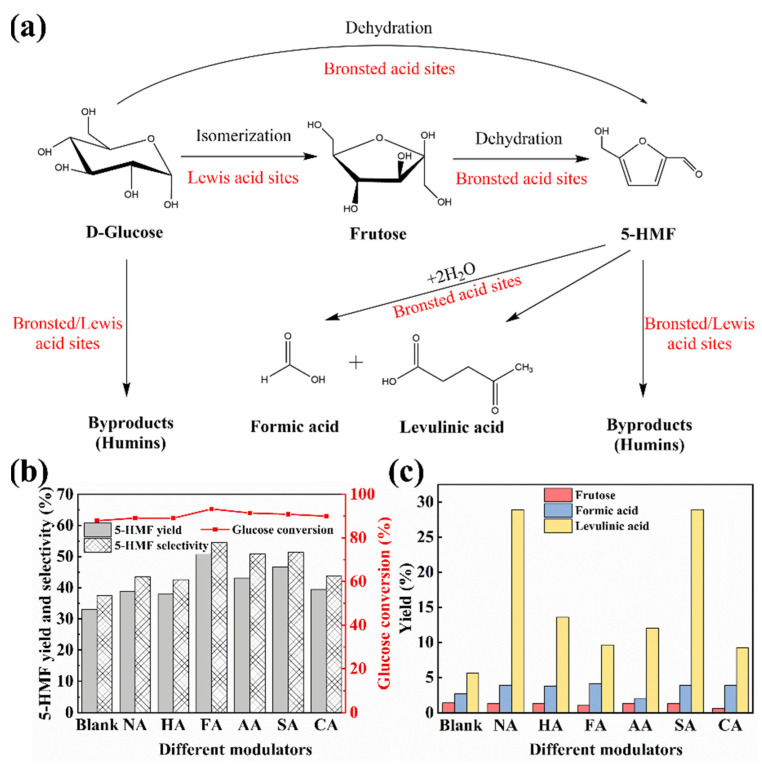
Effects of the blank sample and MIL-101 (Cr, Sn) synthesized with different modulators on glucose conversion to 5-HMF. (**a**) Scheme for synthesizing 5-HMF using glucose as raw material, (**b**) yield, selectivity of 5-HMF, and glucose conversion; (**c**) yields of fructose, formic acid, and levulinic acid.

As shown in Figure 8a, glucose is first isomerized to fructose by the action of Lewis acid, and then three molecules of water are removed from fructose by the action of Bronsted acid to produce 5-HMF [47]. The blank sample and MIL-101 (Cr, Sn) synthesized with different modulators were used as catalysts to investigate their catalytic performance for glucose conversion to 5-HMF. As shown in Figure 8b, the 5-HMF yield was 32.98%, selectivity was 37.54%, and glucose conversion was 87.86% with the blank sample. When NA and HA were used as modulators, the 5-HMF yield was 38.73% and 37.92%, respectively. When AA, SA, and CA were used as modulators, the respective 5-HMF yield was 43.12%, 46.63%, and 39.45%. Finally, when FA was used as a modulator, the 5-HMF yield was 50.79%, selectivity was 54.47%, and glucose conversion was the highest, at 93.24%. The yields of fructose and the by-products FA and LA are shown in Figure 8c. The fructose yields were approximately 1%; however, the MIL-101 (Cr, Sn) catalyzed by NA and SA produced approximately 40% of by-products.

In contrast, the catalytic results of MIL-101 (Cr, Sn) synthesized with modulators increased to different degrees. Thanks to the synergistic effect of its large SSA and pore structure, good contact angle, and average particle size of 30 nm, MIL-101 (Cr, Sn) modulated with FA could react with glucose as much as possible, achieving the highest 5-HMF yield. For the same reason, as a modulator, SA revealed the second largest 5-HMF yield. Besides, MIL-101 (Cr, Sn) with AA and CA showed the fourth and second highest pore size and pore capacity, respectively, with a 5-HMF yield only second to MIL-101 (Cr, Sn) with SA. However, MIL-101 (Cr, Sn) with NA and HA had the smallest pore size and pore capacity, impairing glucose access to the active site for reaction, thus rendering the catalytic performance relatively poor.

## 4. Conclusions

In this study, bimetallic MIL-101 (Cr, Sn) was successfully synthesized using six acids via a modulator-assisted microwave hydrothermal method. PXRD, SEM, and TGA techniques were used to characterize the synthesized particles. The catalytic conversion of glucose to 5-HMF was also comparatively analyzed. The introduction of AA improved the porosity of MOF particles, yielding the highest SSA, pore size, and pore capacity, more satisfactory particle size distribution, and higher chemical and thermal stability. In particular, AA as a modulator produced a higher crystal yield than other acids. In the catalytic glucose conversion to 5-HMF, the yield of 5-HMF was 43.1%, and the conversion of glucose was 91.4%.

## Figures and Tables

**Table 1:** Specific surface area, pore size, and pore volume of the blank sample and MIL-101 (Cr, Sn) with different modulators as an additive.

Different Modulators	BET Surface Area ^1^ (m^2^ g^−1^)	Pore Size ^2^ (nm)	Pore Volume ^3^ (cm^3^ g^−1^)
Blank	2069	0.87	0.48
NA	2426	0.84	0.51
HA	2321	0.76	0.44
FA	2251	0.90	0.50
AA	2504	0.89	0.56
SA	1160	1.00	0.29
CA	2151	0.92	0.50

^1^ Calculated in the pressure range 0.05 < p/p_0_ < 1.0 from N_2_ sorption isotherms at 77 K with an estimated standard deviation of ±50 m^2^ g^−1^. ^2^ Adsorption average pore diameter (4 V/A). ^3^ Single point adsorption total pore volume of pores less than 1.1415 nm width at P/P_0_ = 0.01.

## Data Availability

The data presented in this study are available on request from the corresponding author.

## Supplementary Materials

The following supporting information can be downloaded at: www.mdpi.com/article/10.3390/polym14183826/s1, Figure S1: Diagram for the synthesis studies along with ligand structure and modulators of MIL-101 (Cr, Sn). (**a**) Chromium trimer; (**b**) H_2_BDC; (**c**) super tetrahedron; (**d**) ball-and-stick view of the cages; (**e**) ball-and-stick representation of one unit cell; (**f**) modulator connection. Chromium octahedra, oxygen, and carbon atoms are in green, red, and grey, respectively.; Figure S2: Flow diagram for synthesizing of blank sample and MIL-101 (Cr, Sn) synthesized with different modulators. (**a**–**c**) Blank, (**d**–**f**) nitric acid, (**g**–**i**) hydrochloric acid, (**j**–**l**) formic acid, (**m**–**o**) acetic acid, (**p**–**r**) succinic acid, and (**s**–**u**) citric acid.
