# Supplementary material for: Effects of Acid Modulators on the Microwave-Assisted Synthesis of Cr/Sn Metal-Organic Frameworks
Source: Polymers (Basel). 2022 Sep 13;14(18):3826. doi: 10.3390/polym14183826 (PMC9504004; doi:10.3390/polym14183826)
Supplement: Supplementary file 1 [file polymers-14-03826-s001.zip › polymers-1903957-supplementary.pdf]

polymers

Supplementary Materials: Effects of Acid Modulators on the Microwave-Assisted Synthesis of Cr/Sn Metal-Organic Frameworks

Wei Mao, Renting Huang, Hao Xu, Hao Wang, Yi Huang, Shurong Huang and Jinghong Zhou *

Guangxi Key Laboratory of Clean Pulp & Papermaking and Pollution Control, School of Light Industrial and Food Engineering, Guangxi University, Nanning 530004, China
* Correspondence: jhzhoudou@gxu.edu.cn

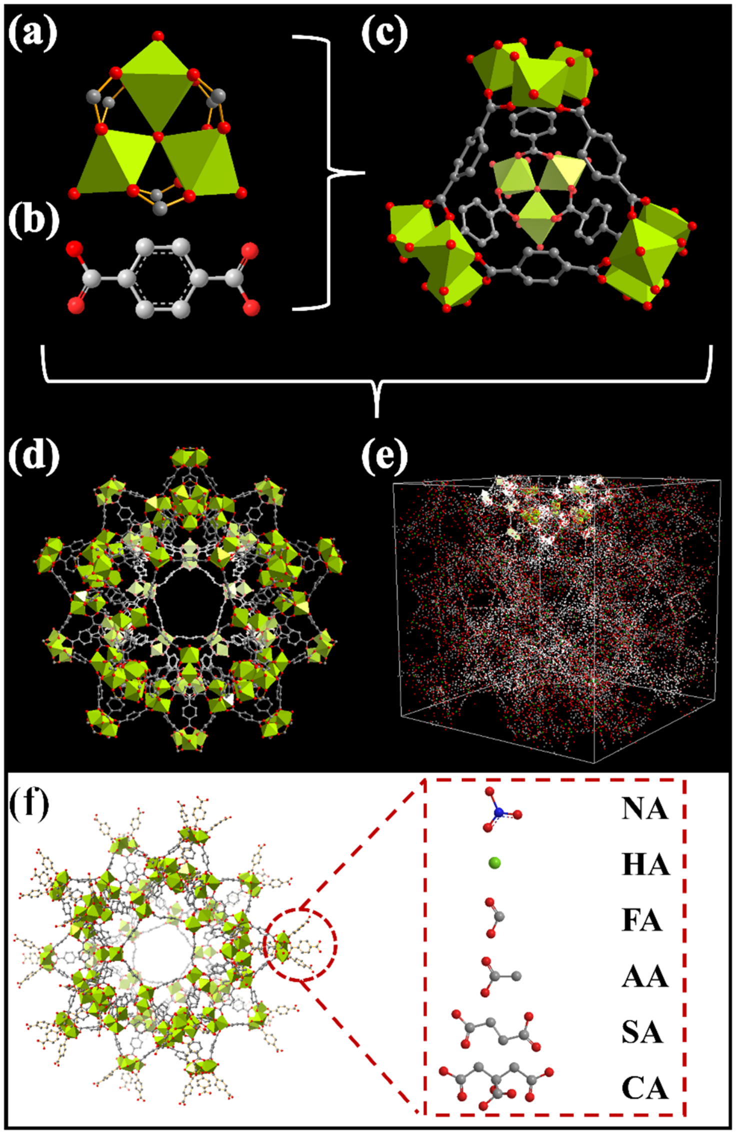

Figure S1. Diagram for the synthesis studies along with ligand structure and modulators of MIL-101 (Cr, Sn). (**a**) Chromium trimer; (**b**) H_2BDC; (**c**) super tetrahedron; (**d**) Ball-and-stick view of the cages; (**e**) ball-and-stick representation of one unit cell; (**f**) modulator connection. Chromium octahedra, oxygen, and carbon atoms are in green, red, and grey, respectively.

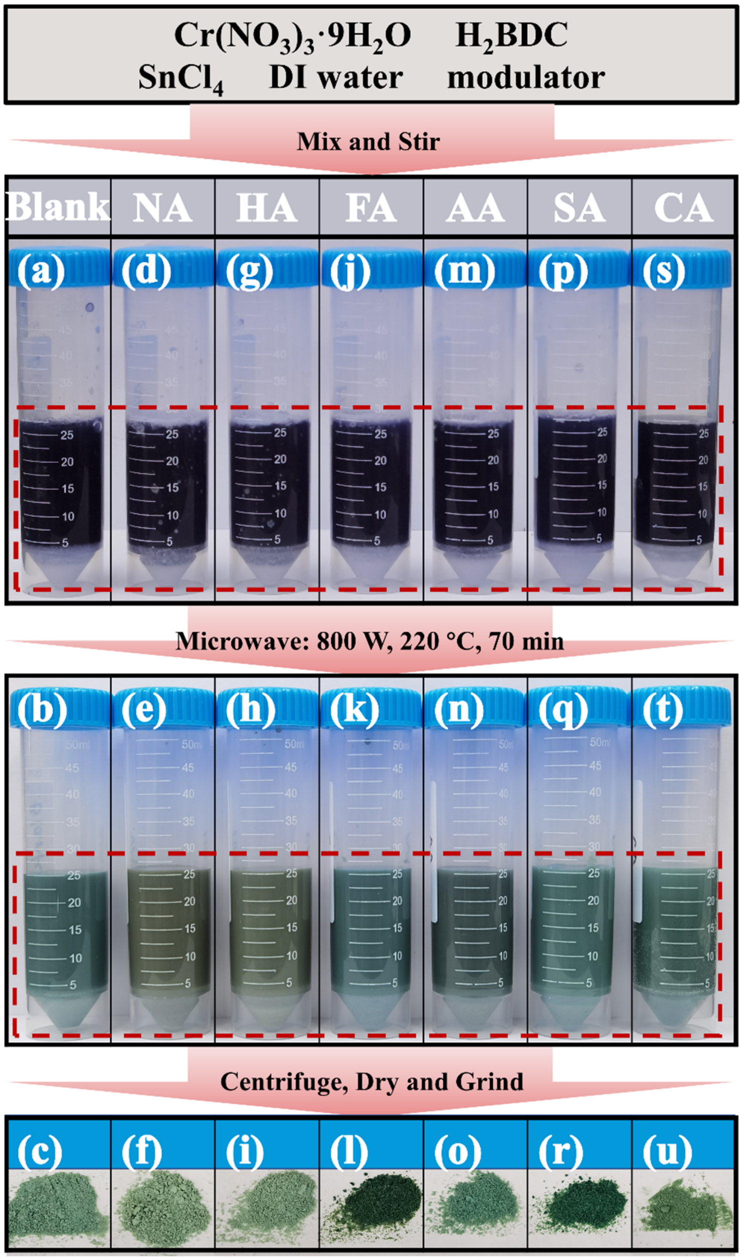

Figure S2. Flow diagram for synthesizing of blank sample and MIL-101 (Cr, Sn) synthesized with different modulators. (**a**–**c**) Blank, (**d**–**f**) nitric acid, (**g**–**i**) hydrochloric acid, (**j**–**l**) formic acid, (**m**–**o**) acetic acid, (**p**–**r**) succinic acid, and (**s**–**u**) citric acid.
